# False positive immunoassay for heparin-induced thrombocytopenia in the presence of monoclonal gammopathy: a case report

**DOI:** 10.11613/BM.2017.030801

**Published:** 2017-10-15

**Authors:** Ivana Markovic, Zeljko Debeljak, Bojana Bosnjak, Maja Marijanovic

**Affiliations:** 1 Institute of Clinical Laboratory Diagnostics, Osijek University Hospital, Osijek, Croatia; 2 Faculty of Medicine, JJ Strossmayer University of Osijek, Osijek, Croatia; 3 Institute of Transfusion Medicine, Osijek University Hospital, Osijek, Croatia

**Keywords:** heparin-induced thrombocytopenia, immunoassay, functional serotonin release assay, cross-reaction, monoclonal gammopathy of undetermined significance

## Abstract

Heparin induced thrombocytopenia (HIT) is a life-threatening disorder which diagnosis depends on laboratory evaluation. The objective of this report is to present the impact of different laboratory methods for HIT detection on the diagnostic evaluation process. In this case, a 78-year old female patient previously diagnosed with monoclonal gammopathy of undetermined significance (MGUS) was administered with heparin for pulmonary embolism treatment. Patient’s initial diagnostic work-up (determination of platelet count and prothrombin time measurement for monitoring of pharmacotherapy) was followed by the clinical estimation of HIT likelihood by “4Ts” score, two immunoassays (ID-PaGIA Heparin/PF4 Antibody Test and ELISA PF4 IgG assay) and one functional test called high-performance liquid chromatography serotonin release assay (HPLC-SRA). The result of “4Ts” score indicated a low likelihood of HIT but persistent thrombocytopenia that appeared days after discontinuation of heparin therapy suggested delayed-onset HIT. Both immunoassays were positive for presence of HIT-autoantibodies, while the functional HPLC-SRA was negative. Since different methods gave opposing results, their interpretation required great attention. In comparison to the HPLC-SRA, immunoassays are prone to the analytical interferences associated with the presence of non-specific antibodies, which may lead to false positive results. In this case, where the patient is known to produce antibodies of undetermined significance, HIT was ruled out as the possible cause of persistent thrombocytopenia primarily due to the negative result of HPLC-SRA, which is not prone to this type of interferences, but also due to the low “4Ts” clinical score.

## Introduction

Heparin-induced thrombocytopenia type II (HIT) is a rare, immune-mediated condition that may emerge in the course of heparin therapy or as a late response reaction. It is caused by an autoantibody directed against heparin/platelet factor 4 (PF4) complex. New heparin/PF4/antibody complex attaches to the platelet surface FCγIIa receptors causing platelet activation, aggregation and increased clearance that lead to serious thrombocytopenia (*1*-*5*). Simultaneously, platelet activation initiates the blood-coagulation cascades often resulting in the thrombotic events, which may be lethal.

A straightforward, yet not definitive approach to the determination of the clinical likelihood of HIT relies on several scoring systems, among which “4Ts” and HIT Expert Probability (HEP) scores are the most frequently used. The “4Ts” score takes into account the magnitude of thrombocytopenia, timing of onset of thrombocytopenia, thrombosis and other causes of thrombocytopenia. On the other hand, HEP score considers the magnitude and timing of fall in platelet count, nadir platelet count, thrombosis, skin necrosis, acute systemic reaction, bleeding and other causes of thrombocytopenia (*2*, *6*). Inconclusive scores are followed by laboratory HIT evaluation that includes immunoassays and/or tests of platelet function. In immunoassays, such as enzyme linked immunosorbent assay (ELISA), particle gel immunoassay (PaGIA) and particle immunofiltration assay (PIFA), HIT-autoantibodies bind to the PF4/heparin or similar complex attached to the solid phase or dissolved in the liquid phase (*2*-*4*, *6*, *7*). The ability of HIT-autoantibody to activate healthy donor platelets in presence of heparin is the basis of functional tests like serotonin release assays (SRA) (C^14^-SRA, high performance liquid chromatography (HPLC)-SRA, flow cytometry SRA) and platelet aggregation assays (heparin induced platelet activation assays (HIPA) and platelet aggregation test (PAT)) (*2*-*4*, *6*, *7*).

Immunoassays are characterized by high sensitivity (89-97%) and high negative predictive value (> 95%) but the tendency to nonspecific antibody binding lowers diagnostic specificity of HIT immunoassays to 80-90% (*6*-*9*). Potential false positive results can occur in conditions and diseases with the excessive production of antibodies, like in autoimmune diseases and some hematologic disorders. Monoclonal gammopathy of undetermined significance (MGUS) is such a condition, where elevated production of monoclonal antibodies by plasma cells may lead to cross-reactions with the immunoassay test components (*1*). Regarding that, Nazi *et al*. presented results of HIT testing in a large method comparison study, where 20.9% of samples were immunoassay positive, but functional test results were negative (*10*). In comparison to immunoassays, functional tests have higher specificity and sensitivity (both > 95%) since they detect only a subset of antibodies that activate platelets in heparin-dependent manner (*6*, *7*, *11*). The exception is PAT, the sensitivity of which is lower due to application of platelet rich plasma rather than washed platelets (*6*). The aim of this case report is to present the impact of different laboratory methodologies on the establishment of HIT diagnosis in the presence of MGUS. To the best of our knowledge, this is the first such case ever reported.

## Materials and methods

### Subject

Here we present a case of a 78-year old female patient admitted to Emergency Department of Osijek University Hospital (Osijek, Croatia) with the breathing difficulties. Her medical history included hypertension, diabetes mellitus type II, hyperuricemia, MGUS IgG lambda (λ) type. Computer tomography scan of the patient’s thorax showed pulmonary embolism that was treated by low molecular weight heparin (LMWH). LMWH therapy has been administrated from the day of admission (day 1) to the 10th day of hospitalization. On the 4th day of hospitalization, oral anticoagulant therapy was introduced. After 13 days the patient was discharged from the hospital. She has been instructed to continue with the coumarin therapy.

Two weeks later the patient was readmitted to Emergency Department of Osijek University Hospital with mouth bleeding and hematomas on her abdomen, arms and legs. This time the patient was diagnosed with severe thrombocytopenia and oral anticoagulant overdose which were managed at admission by a dose of vitamin K, platelet concentrate and corticosteroids. The bleeding stopped. Due to severe thrombocytopenia and a history of recent heparin therapy, the delayed onset of HIT was suspected. All anticoagulant therapy was immediately ceased and new thrombotic incident did not occur. As the patient already suffered from thrombocytopenia caused by angiotensin-converting-enzyme inhibitor approximately one year before this event and then responded well to corticosteroid therapy, the same therapy was applied on day 2 during the second hospitalization. However, 14 days later, the thrombocytopenia persisted and immunoglobulin and rituximab therapy was started. After 37 days, patient was discharged with persistent thrombocytopenia.

Signed informed consent was obtained from the patient. Diagnostic work-up was performed in accordance with the Declaration of Helsinki.

### Sample collection and routine evaluation

Patient’s whole blood samples and plasma samples were collected in K_3_-EDTA tubes and tubes containing 0.105 M sodium citrate, respectively (Becton Dickinson, Franklin Lakes, USA). Throughout patient’s first and second hospitalization platelet count was determined by the Sysmex 2000XN automated haematology analyser (Sysmex Corporation, Kobe, Japan) while the prothrombin time was measured by the BCS XP automated coagulation analyser (Siemens Healthcare, Erlangen, Germany). On day 2 of the second hospitalization patient’s blood for HIT-autoantibodies detection was sampled in tubes containing no additives (Becton Dickinson, Franklin Lakes, USA). The diagnostic algorithm for the evaluation of suspected HIT was performed during the second hospitalization in accordance with Farm *et al*. (*11*). On the day after admission (day 2), clinical likelihood of HIT was assessed by the clinician according to the “4Ts” score questionnaire (*2*, *6*). A total score of 0-3 is associated with low likelihood, 4-5 is associated with medium and 6-8 stands for a high clinical likelihood of HIT. Score calculation and two immunoassays were performed on the same day, while the functional test was performed on day 4.

### Gel agglutination assay

Gel agglutination assay was the first immunoassay used for the diagnostic evaluation of our subject. Rapid particle gel agglutination immunoassay, namely ID-PaGIA Heparin/PF4 Antibody Test (Diamed SA, Cressier sur Morat, Switzerland) is a qualitative/semi-quantitative test that uses high density polymeric particles coated with the PF4/heparin complex deposited on a gel matrix. According to the manufacturer, positive reaction can be graded as: complete agglutination on the top of the gel (grade 4+); strong agglutination with agglutinates distributed within upper part of the gel (grade 3+); partial agglutination when some particles reach the bottom of the microtube with agglutinates still visible in the upper part of the gel (grade 2+), agglutination throughout the gel (grade 1+ to 2+). The lesser agglutinations are considered as doubtful reactions. In the absence of HIT-autoantibodies *i.e.* negative reaction, all particles are compacted at the bottom of the test microtube, otherwise the test is considered positive or doubtful. The test performance is described in Pouplard *et al.* (*12*).

### PF4 IgG ELISA

Gel agglutination assay was followed by the PF4 IgG ELISA. Qualitative/semi-quantitative ELISA PF4 IgG assay (Immucor GTI Diagnostics, Waukesha, USA) uses microwells coated with polyvinyl sulfonate (PVS) complexed with PF4. According to the manufacturer, test results showing optical density (OD) values equal or greater than 0.400 are regarded as positive for presence of HIT-autoantibodies. The test was performed according to the manufacturer instructions described in Whitlatch *et al*. (*13*).

### HPLC-SRA assay

**Figure 1:**
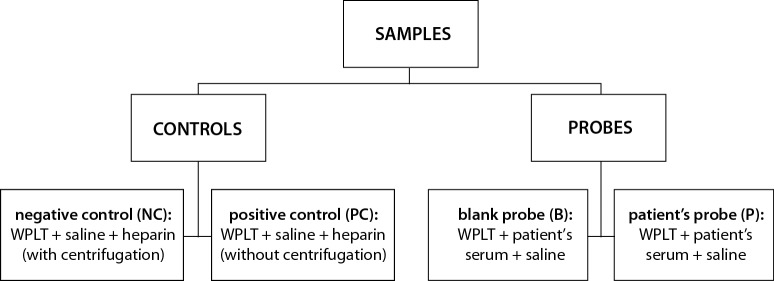
Sample preparation flow chart. WPLT – washed platelets.

A functional test, in this case HPLC-SRA, was used for confirmation of HIT diagnosis. In the presence of HIT-autoantibodies, platelets undergo activation and degranulation followed by the serotonin release. Platelets were acquired from four donors with 0 negative blood type and platelet concentrate was prepared in house in accordance with the European Directorate for the Quality of Medicines and Healthcare good practice guidelines (*14*). Platelet count and mean platelet volume (MPV) of unwashed concentrate were determined by the Sysmex 2000XN automated haematology analyser (Sysmex Corporation, Kobe, Japan). Platelet washing was done according to the procedure proposed by Debeljak (*15*). After washing, platelet count and MPV were determined again. The system suitability criteria were platelet count in the range of 250-450 x10^9^/L and MPV alteration less than 10% of its initial value. Positive and negative control samples and patient’s blank and probe samples were prepared according to Koch *et al.* (Figure 1) (*16*).

Commercial IVD ClinRep HPLC Serotonin in Plasma validated kit (Recipe Chemicals + Instruments GmbH, Munich, Germany) was used for quantification of the released serotonin (*17*). The measurement was conducted using Shimadzu Class VP 10 series (Shimadzu, Kyoto, Japan) HPLC instrument equipped with the electrochemical detector (ECD) CLC 100 (Chromsystems, Gräfelfing, Germany). Serotonin standard, internal standard solutions and analytical column were acquired from the kit manufacturer. Sample injection volume was set to 10 µL, flow rate was set to 1.0 mL/min, run time was set to 8 minutes and applied voltage on ECD was set to 0.6 V. The exact procedure is described elsewhere (*15*).

Percentage of serotonin release (SR) from functional HPLC-SRA was calculated according to the following equation: SR = P_R_ / PC_R_ x 100%, where P_R_ stands for serotonin peak area and internal standard peak area ratio for patient’s probe. PC_R_ stands for serotonin peak area and internal standard peak area ratio for positive control. If SR exceeds 20%, the patient is considered positive for presence of HIT-autoantibodies.

## Results

Platelet counts and prothrombin times determined during the first and the second hospitalization are given in Tables 1 and 2. The patient’s “4Ts” score of 3 points signifies low likelihood of HIT, where 1 point was accounted for the platelet count fall 30-50% or platelet nadir 10-19 x10^9^/L, 1 point was accounted for the onset that took place after day 10 of therapy (prior heparin exposure 30-100 days ago) and 1 point was accounted for other possible causes for thrombocytopenia.

**Table 1:** Pharmacotherapy, platelet counts and prothrombin times during first hospitalization

	**Day 1**	**Day 2**	**Day 3**	**Day 7**	**Day 8**	**Day 10**	**Day 13**	**Reference interval**
**Platelet count (x10^9^/L)**	279	220	326	261	247	224	NA	158 - 424
**Prothrombin time ratio (%)**	NA	NA	0.71	0.18	0.34	0.30	0.34	0.70 – 1.27
**Prothrombin time INR**	NA	NA	1.17	3.21	1.97	2.16	1.98	/
**LMWH therapy**	+	+	+	+	+	+	-	/
**Cumarin anticoagulant therapy**	-	-	-	+	+	+	+	/
LMWH – low molecular weight heparin. NA - not available. (+) - Present. (-) - Absent.								

**Table 2:** Pharmacotherapy, platelet counts and prothrombin times during second hospitalization

	**Day 1**	**Day 3**	**Day 4**	**Day 5**	**Day 19**	**Day 34**	**Day 37**	**Reference interval**
**Platelet count (x10^9^/L)**	13	14	12	8	24	19	22	158 - 424
**Prothrombin time ratio (%)**	0.06	0.81	0.75	0.89	1.14	1.01	NA	0.70 – 1.27
**Prothrombin time INR**	> 6.0	1.12	1.15	1.07	0.95	0.99	NA	/
**LMWH therapy**	-	-	-	-	-	-	-	/
**Cumarin anticoagulant therapy**	+	-	-	-	-	-	-	/
LMWH – low molecular weight heparin. NA - not available. (+) - Present. (-) - Absent.								

**Figure 2:**
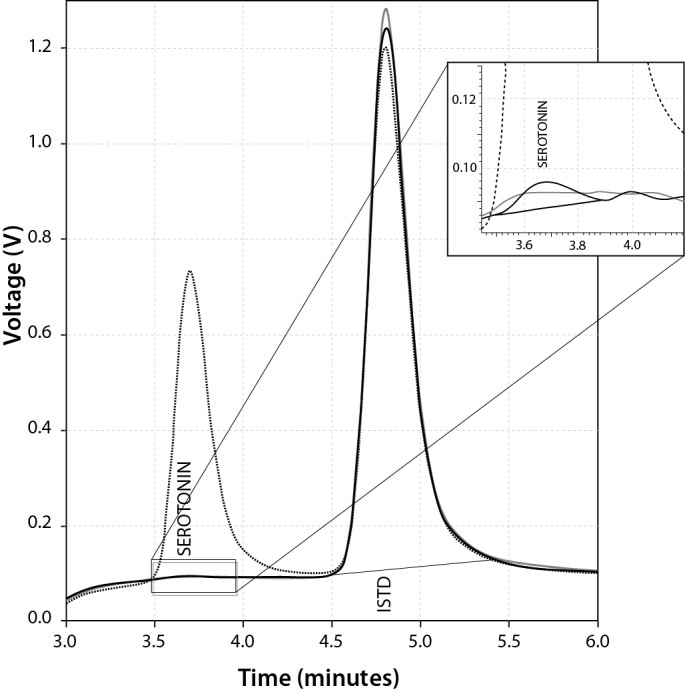
Serotonin and internal standard (ISTD) peak in HPLC-SRA. The solid line corresponds to the patient’s probe, the dotted line to the positive control and grey line to the blank probe.

According to the rapid assay ID-PaGIA Heparin/PF4 Antibody Test patient’s serum was positive for HIT-autoantibodies (grade 3+). ELISA PF4/IgG Assay also gave positive result: OD value was 1.28. In contrast to the immunoassays, functional HPLC-SRA was negative. Serotonin release for the patient’s sample calculated according to the equation was 1.5% (Figure 2).

## Discussion

HIT is strongly suspected in patients on heparin therapy if platelet count drops under 150 x10^9^/L (median platelet count nadir is about 55 x10^9^/L) and if platelet count declines for more than 50% from its initial value (*2*, *3*). During pharmacotherapy with the LMWH, patient’s platelet count was within reference range (Table 1), but the platelet count decrease and the coumarin overdose were registered 14 days after discontinuation of therapy (Table 2). Although “4T”s score indicated low likelihood of HIT, low platelet count was consistent with delayed-onset HIT. According to the literature, sera from patients with delayed-onset are usually strongly reactive in the immunoassays and activate platelets in SRA even in the absence of pharmacologic heparin concentrations (*18*, *19*). With that in mind, we performed a laboratory evaluation without which possible HIT diagnosis cannot be confirmed or rejected.

The first test we used was gel agglutination test, which is widely available due to its low cost, simplicity of performance and low time consumption. ID PaGIA test was highly positive (grade 3+), but the interpretation of test result is susceptible to subjective reading. To improve diagnostic certainty, we performed ELISA IgG, a test characterized by automated, *i.e.* less subjective, measurement. The OD value of 1.28 also suggested presence of HIT-autoantibodies and was consistent with delayed-onset HIT. In compliance with our patient’s immunoassay test results and published research results we expected moderately strong donor platelet activation with patient’s sera in HPLC-SRA test (*20*). Contrary to that, the percentage of released serotonin was low and diagnosis of HIT was excluded.

All immunoassays share a common interference in the form of cross-reactivity and unspecific binding that may lead to the false positive results (*21*). Presence of the MGUS IgG λ endogenous antibodies in patient’s serum raises a suspicion that these antibodies non-specifically bind to the PF4/heparin in gel agglutination assay and to the PF4/PVS in ELISA causing positive reaction in both tests. The manufacturer of the ELISA PF4 IgG assay clearly states that the presence of immune complexes or other immunoglobulin aggregates in patient’s sample may cause an increased nonspecific binding leading to the false-positive results. Along with that, the literature describes several cases where HIT was misdiagnosed or over diagnosed due to interferences and cross-reactivity in immunoassays. For example, Hron *et al*. described two cases where immunoassays were false positive for the presence of HIT-autoantibodies (*22*). In the first case, an elderly patient developed alloantibodies to human platelet antigen-1a as a consequence of blood transfusion which led to thrombocytopenia and post-transfusion purpura. In the second case, an elderly patient developed the piperacilin-dependent platelet antibodies and nonpathogenic heparin/PF4 IgA antibodies, which led to the drug-induced thrombocytopenia after pneumonia treatment with piperacilin/tazobactam. The functional test in the second case was negative. Moreover, Alpert *et al.* found the prevalence of heparin/PF4 antibodies ranging between 4 and 15% in patients with antiphospholipid syndrome and systemic lupus erythematosus, respectively, often in the absence of clinical HIT or recent heparin exposure (*23*). As previous cases demonstrate, leaning on positive immunoassay alone can lead to a wrong suspicion about HIT and potential use of other anticoagulant drugs that can cause bleeding and additional healthcare costs (*24*-*26*). For that and similar reasons samples positive for HIT-autoantibodies in immunoassays should be evaluated by the functional tests.

HPLC-SRA is a modified version of the gold standard SRA-^14^C test but it does not require usage of radioactive material. The test has its own disadvantages like the requirement for the expensive equipment, requirement for a high level of expertise and the long duration of sample preparation. Other limitations of the functional tests include selection of donor platelets due to their significant interindividual variability in activation responsiveness (*4*, *27*) and non-standardized analysis of serotonin release which is, in this case, an in-house solution. As HPLC-SRA test is characterized by considerably higher sensitivity and specificity than immunoassays, it served as a definitive test in this study. To recapitulate, the correct sequence of HIT diagnostic evaluation should follow the these steps: 1) assessment of the HIT clinical likelihood with the “4Ts” or other score questionnaire, 2) screening for possible HIT autoantibodies with accessible immunoassays, and 3) application of the HPLC-SRA or other highly specific assay, when the immunoassay results are positive or doubtful, for conformation or rejection of the HIT.

Based on HPLC-SRA, HIT has been excluded as an unlikely diagnosis but the cause of thrombocytopenia in this patient remained unknown. As shown in Table 2, the thrombocytopenia was still present after discontinuation of anticoagulant therapy, which leads to suspicion of immune thrombocytopenia, a state that may appear in patients with MGUS (*28*, *29*). However, absence of a serotonin peak in the blank probe in HPLC-SRA (Figure 2) demonstrates that the MGUS or antibodies other than HIT autoantibodies did not activate donor platelets. In addition, patient did not respond to corticosteroid, rituximab and immunoglobulin therapy during her second hospitalization, which implies that cause of the thrombocytopenia most likely, was not immune.

In conclusion, laboratory evaluation of HIT requires great caution. In comparison with HPLC-SRA, immunoassays are easier to perform but the presence of the non-specific antibodies may lead to the false positive results. Because of high sensitivity and high negative predictive value, immunoassays are appropriate tools for ruling out the HIT diagnosis. HPLC-SRA and related functional tests are more sensitive and more specific. For the confirmation of HIT diagnosis and differential diagnostics of thrombocytopenia described functional tests should be applied.
